# *PRSS3*/Mesotrypsin and kallikrein-related peptidase 5 are associated with poor prognosis and contribute to tumor cell invasion and growth in lung adenocarcinoma

**DOI:** 10.1038/s41598-018-38362-0

**Published:** 2019-02-12

**Authors:** Honghai Ma, Alexandra Hockla, Christine Mehner, Matt Coban, Niv Papo, Derek C. Radisky, Evette S. Radisky

**Affiliations:** 1 Department of Cancer Biology, Mayo Clinic, Jacksonville, FL 32224 USA; 2 Department of Thoracic Surgery, First Affiliated Hospital, School of Medicine, Zhejiang University, Hangzhou, 310003 China; 3 Department of Biotechnology Engineering and the National Institute of Biotechnology in the Negev, Ben-Gurion University of the Negev, Beer-Sheva, Israel

## Abstract

Serine proteases have been implicated as key drivers and facilitators of lung cancer malignancy, and while these proteins represent straightforward targets for therapeutic inhibitors, identification of optimal points for intervention has been complicated by the complex networks in which these enzymes function. Here we implicate a signaling pathway consisting of PRSS3/mesotrypsin and kallikrein-related peptidase 5 (KLK5) in lung adenocarcinoma malignancy. We show that elevated PRSS3/mesotrypsin expression is prognostic for poor outcome for patients with lung adenocarcinoma, and that genetic or pharmacologic targeting of PRSS3/mesotrypsin reduces lung adenocarcinoma cell invasiveness and proliferation. We further show that genetic targeting of KLK5, a known target of PRSS3/mesotrypsin, phenocopies the effect of PRSS3/mesotrypsin knockdown, and also that elevated expression of KLK5 is similarly prognostic for outcome in lung adenocarcinoma. Finally, we use transcriptional profiling experiments to show that PRSS3/mesotrypsin and KLK5 control a common malignancy-promoting pathway. These experiments implicate a potential PRSS3/mesotrypsin-KLK5 signaling module in lung adenocarcinoma and reveal the potential therapeutic benefit of selectively targeting these pathways.

## Introduction

Lung cancer is responsible for the greatest number of cancer deaths in the U.S. for both men and women, with 234,000 new cases and 154,000 deaths estimated in 2018^1^. The 5-year survival rate is 18%, declining to 5% when distant metastasis is present at diagnosis, as is the case in a majority of patients^1^. Lung cancers comprise two main types, small cell lung cancer (SCLC) and non-small cell lung cancer (NSCLC), accounting for 15% and 85%, respectively^2^; NSCLC is further divided among lung adenocarcinoma (LAC, 50%), squamous cell carcinoma (SCC, 30%), and others (20%)^3^. The past decade has seen a major shift in the treatment paradigm for NSCLC, toward targeted therapies guided by mutation and biomarker-based stratification^3–6^. Nevertheless, around 40% of NSCLCs carry no known driver mutation, and even for those with targetable mutations the response to therapies such as tyrosine kinase inhibitors is often short-lived^3,6^; thus, there remains a compelling need to unravel mechanisms of disease progression to identify new targets and strategies for treatment.

Extracellular proteases represent established and emerging drivers of tumorigenesis and tumor progression, and may offer useful therapeutic targets in lung cancer and other cancers^7^. The serine proteases in particular include many secreted and cell membrane associated enzymes that become dysregulated in cancer and can contribute to multiple aspects of tumor progression^8–14^. These proteases often function not in isolation, but can act cooperatively in signaling cascades or complex regulatory networks, sometimes spanning multiple protease families and classes, a concept that has been referred to as the “protease web”^15^. One protease may activate others by proteolytic processing of pro-enzyme precursors, or may influence the catalytic activity of other proteases through cleavage and inactivation of endogenous protein protease inhibitors. An exemplar of the latter mechanism is offered by mesotrypsin; this isoform of the digestive protease trypsin has evolved novel catalytic features enabling it to proteolytically inactivate many endogenous human protease inhibitors that regulate other serine proteases^16–19^. Given this unusual capability, mesotrypsin may influence the activity *in vivo* of a wide variety of serine proteases, thus representing a regulatory node in the protease web^16,17^.

Mesotrypsin, encoded by the *PRSS3* gene, has been strongly implicated in tumor growth and metastatic progression of cancers including prostate cancer and pancreatic cancer^20,21^. In prostate cancer experimental studies, knockdown of PRSS3/mesotrypsin expression inhibited anchorage independent growth and invasion of cancer cells, and suppressed metastasis in orthotopic mouse models^20^. Likewise in pancreatic cancer experimental studies, overexpression of PRSS3/mesotrypsin promoted cancer cell proliferation, invasion and metastasis, while knockdown of endogenous PRSS3/mesotrypsin reduced these malignant phenotypes^21^. While the role of mesotrypsin in lung cancer has not been as well-studied, a transcriptional profiling study identified *PRSS3* as one of several genes predictive of future distant metastasis and poor survival when expressed in early stage NSCLC tumors^22^. When overexpressed in a SCC cell line, a PRSS3-derived fusion protein led to increased migration of the cancer cells through an endothelial cell layer, suggesting a potential role for PRSS3/mesotrypsin in metastatic dissemination^22^.

In the present study, we identify *PRSS3* gene expression as a prognosticator of poor survival and cancer progression specifically in LAC but not in SCC. Using an LAC-derived cell line with high endogenous expression of *PRSS3*, we show that silencing of *PRSS3* gene expression, or inhibition of mesotrypsin activity, suppresses cancer cell growth and invasion, implicating mesotrypsin as a driver of malignancy in LAC. Finally, we identify the serine protease kallikrein 5 as a potential mediator in the protease network influenced by mesotrypsin; these two proteases are found to regulate a common, distinctive gene signature responsible for malignant behavior in LAC.

## Results

### *PRSS3* is prognostic of poor survival and cancer progression in lung adenocarcinoma

**Figure 1:**
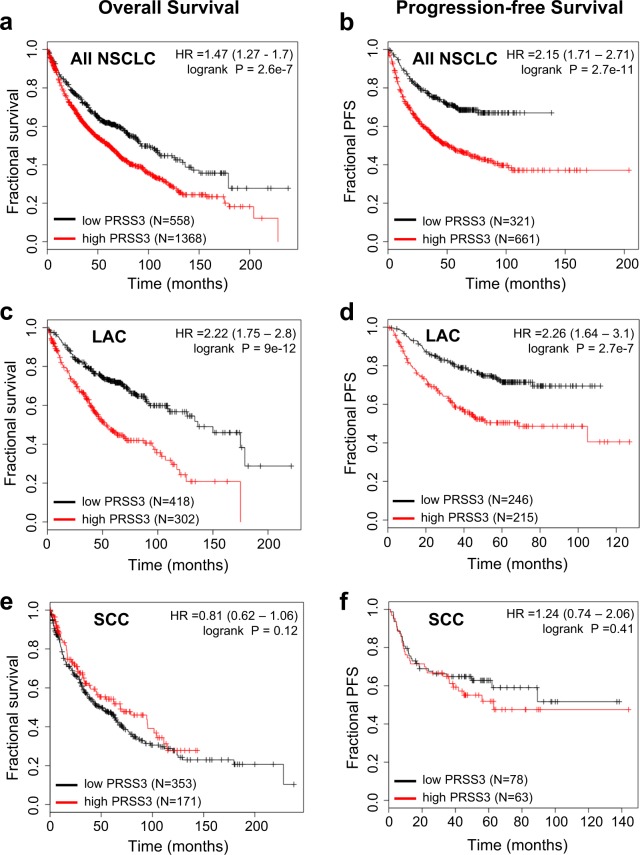
*PRSS3* is prognostic of poor survival and cancer progression in lung adenocarcinoma (LAC) but not squamous cell carcinoma (SCC). Univariate Kaplan-Meier survival analyses of NSCLC patients are plotted, stratified by *PRSS3* mRNA expression. (**a**,**b**) High *PRSS3* expression was significantly associated with (**a**) poor OS and (**b**) poor PFS in analyses including all NSCLC patients. (**c**,**d**) Analyses restricted to patients with LAC also showed highly significant association of *PRSS3* expression with (**c**) poor OS and (**d**) poor PFS, and revealed greater hazard ratios. (**e**,**f**) Analyses of the subset of patients with SCC did not show significant association of *PRSS3* expression with clinical outcomes. Analyses were conducted using the KM Plotter online tool (http://kmplot.com/analysis/)^23^ and included data pooled from 13 cohorts; additional details are described in the Materials and Methods.

To assess the potential association of *PRSS3* gene expression with outcome measures in NSCLC, we conducted a meta-analysis of published lung cancer datasets using the KM Plotter online tool^23^. This analysis used combined data from 13 different microarray gene expression studies with published clinical characteristics including overall survival (OS) and/or progression-free survival (PFS), and included 2435 patients. In analyses including all NSCLC patients, *PRSS3* gene expression showed highly significant association with both poor OS (HR 1.47, P = 2.6e-7; Fig. 1a) and poor PFS (HR 2.15, P = 2.7e-11; Fig. 1b). Limiting the analysis to the subset of patients with lung adenocarcinoma (LAC) resulted in greater hazard ratios relating *PRSS3* expression with poor OS (HR 2.22, P = 9e-12; Fig. 1c) and poor PFS (HR 2.26, P = 2.7e-7; Fig. 1d). By contrast, *PRSS3* showed no significant associations with outcome in the subset of patients with lung squamous cell carcinoma (SCC) (Fig. 1e,f). Further analyses found significant associations of *PRSS3* with poor outcome in all LAC subsets, including men and women, smokers and nonsmokers, and patients stratified by stage (Table 1). We further found the association of *PRSS3* expression with poor survival to be strengthened in multivariate analysis when adjusting for stage (Table 2), identifying *PRSS3* expression as an independent prognostic factor for LAC survival.

**Table 1:** Association of *PRSS3* expression with outcome in lung cancer subsets.

Patient subset	Endpoint	N	HR	CI	P
all	OS	1926	1.47	1.27–1.70	2.60E-07
LAC	OS	720	2.22	1.75–2.80	9.00E-12
SCC	OS	524	0.81	0.62–1.06	1.20E-01
LAC, women	OS	318	2.45	1.63–3.68	8.00E-06
LAC, men	OS	344	2.14	1.54–2.98	3.70E-06
LAC, smokers	OS	246	2.51	1.53–4.14	1.80E-04
LAC, never smoked	OS	143	4.1	1.82–9.24	2.30E-04
LAC, stage 1	OS	370	2.92	1.98–4.31	1.50E-08
LAC, stage 2	OS	136	2.72	1.62–4.58	8.70E-05
all	PFS	982	2.15	1.71–2.71	2.70E-11
LAC	PFS	461	2.26	1.64–3.10	2.70E-07
SCC	PFS	141	1.24	0.74–2.06	4.10E-01
LAC, women	PFS	235	2.53	1.60–3.99	3.50E-05
LAC, men	PFS	226	2.16	1.39–3.36	4.90E-04
LAC, smokers	PFS	243	2.46	1.56–3.90	6.90E-05
LAC, never smoked	PFS	143	2.19	1.18–4.06	1.10E-01
LAC, stage 1	PFS	283	2.71	1.68–4.37	2.10E-05
LAC, stage 2	PFS	103	1.67	0.96–2.90	6.50E-02

LAC, lung adenocarcinoma; SCC, squamous cell carcinoma; OS, overall survival; PFS, progression-free survival, HR, hazard ratio; CI, 95% confidence interval.

**Table 2:** Multivariate analysis of *PRSS3* expression and stage versus overall survival in lung adenocarcinoma.

	HR	CI	P
Stage	1.73	1.43–2.08	<1E-16
*PRSS3* expression	2.42	1.81–3.24	<1E-16

N = 534. HR, hazard ratio; CI, 95% confidence interval.

### *PRSS3* expression promotes invasion and growth of lung adenocarcinoma cells

**Figure 2:**
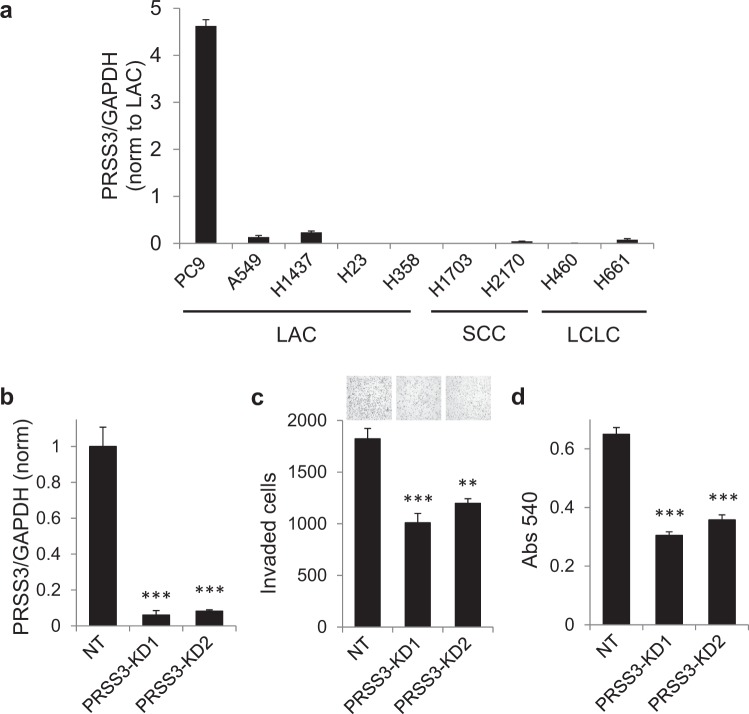
*PRSS3* silencing inhibits invasion and growth of PC9 lung adenocarcinoma cells. (**a**) Baseline expression of PRSS3, determined by qRT/PCR normalized to GAPDH, is plotted for a panel of lung cancer cell lines. Expression levels are normalized to the average for LAC-derived cell lines. LAC, lung adenocarcinoma; SSC, squamous cell carcinoma; LCLC, large cell lung carcinoma. (**b**) *PRSS3* expression is effectively suppressed by two targeted lentiviral shRNA constructs, PRSS3-KD1 and PRSS3-KD2, relative to cells transduced with the non-target control virus (NT), as assessed by qRT/PCR normalized to GAPDH expression. (**c**) Knockdown of *PRSS3* significantly inhibits PC9 cellular invasion in Matrigel transwell assays. Representative images of the invasion assay are shown above each condition. (**d**) Knockdown of *PRSS3* significantly inhibits PC9 cell growth as assessed by MTT assays. Error bars show SEM. ^**^P ≤ 0.01; ^***^P ≤ 0.001.

To assess whether expression of *PRSS3* may play a functional role in promoting malignant progression of LAC, we first examined *PRSS3* expression in a small panel of established lung cancer cell lines. We found that the LAC-derived PC9 cell line showed substantially elevated *PRSS3* transcript expression relative to all other cell lines evaluated (Fig. 2a), and thus could offer a relevant model for high-*PRSS3* LAC. To evaluate the impact of silencing tumor cell *PRSS3* expression in this model, PC9 cells were stably transduced either with a nontarget control (NT) virus recognizing no human genes, or with one of two lentiviral shRNA knockdown constructs specifically targeting the *PRSS3* gene. Efficient *PRSS3* knockdown was achieved as assessed by qRT/PCR (Fig. 2b; Western blot validation, Supplemental Fig. 1a). We next measured the impact of *PRSS3* knockdown on the invasive potential of the cells in Matrigel transwell assays, finding that cells in which *PRSS3* expression was suppressed showed significantly reduced capacity for invasion through artificial basement membrane (Fig. 2c). Finally, we measured the impact of *PRSS3* knockdown on cell growth of PC9 cells by using the MTT assay, which provides a comparative measure of the number of viable and metabolically active cells, reflecting the balance of cell proliferation and cell death. Cells in which *PRSS3* expression was suppressed showed significantly reduced growth in this assay (Fig. 2d). By contrast, overexpression of PRSS3 in the low-expressing cell line H23 led to increased proliferation (Supplemental Fig. 2a,b).

### Inhibition of mesotrypsin activity reduces invasion and proliferation of lung adenocarcinoma cells

**Figure 3:**
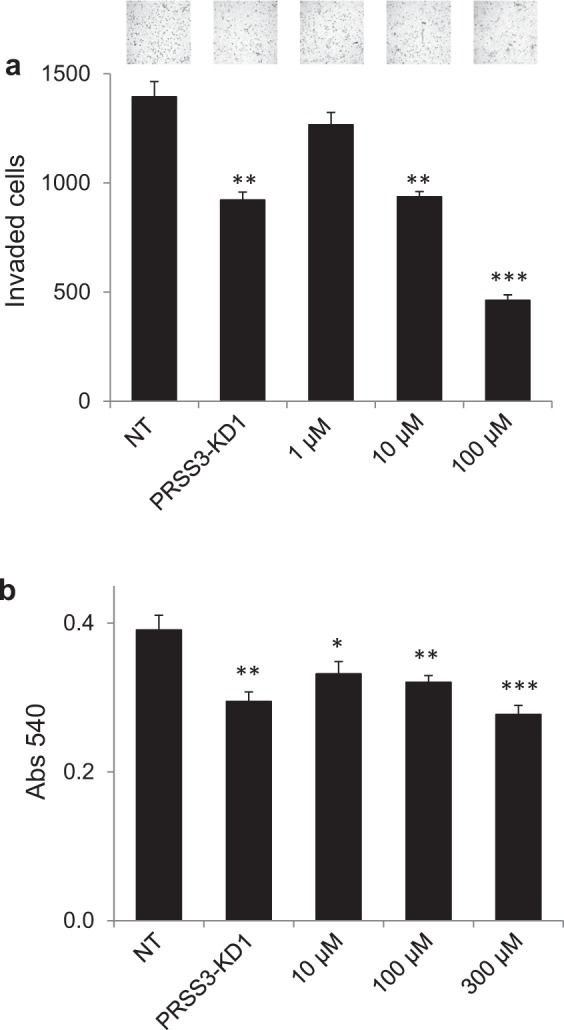
Mesotrypsin inhibition suppresses invasion and growth of PC9 lung adenocarcinoma cells. (**a**) Treatment of PC9 cells with mesotrypsin inhibitor diminazene over a range of concentrations (shown below the x-axis of the graph) significantly inhibited PC9 cellular invasion in Matrigel transwell assays. Cells in which *PRSS3* expression was silenced by shRNA construct PRSS3-KD1 were evaluated for comparison. Representative images of the invasion assay are shown above each condition. (**b**) Treatment of PC9 cells with mesotrypsin inhibitor diminazene over a range of concentrations (shown below the x-axis of the plot) significantly inhibited PC9 cell growth as assessed by MTT assays. Cells in which *PRSS3* expression was silenced by shRNA construct PRSS3-KD1 were evaluated for comparison. Error bars show SEM. ^*^P ≤ 0.05; ^**^P ≤ 0.01; ^***^P ≤ 0.001.

The *PRSS3* gene encodes several splice isoforms of trypsinogen, each of which can become proteolytically activated to produce the identical active protease mesotrypsin. Hypothesizing that the functional significance of *PRSS3* expression in lung adenocarcinoma derives from the proteolytic activity of active mesotrypsin, we next tested the ability of pharmacological inhibitors of mesotrypsin to attenuate PC9 cell invasion and proliferation. In a recent study, we identified the small molecule diminazene as an inhibitor of mesotrypsin activity with an inhibitory constant (*K*_*i*_) of 3.6 µM^24^. Using this inhibitor over a range of concentrations spanning the *K*_*i*_ value, we conducted Matrigel transwell assays to measure cellular invasion and MTT assays to provide a proxy measurement for cell growth of PC9 LAC cells. In parallel we assessed the impact of *PRSS3* knockdown on these phenotypic endpoints. We found a concentration-dependent inhibitory effect for diminazene in invasion assays (Fig. 3a). A concentration of 10 µM diminazene (roughly 3-fold *K*_*i*_) significantly inhibited invasion to a similar extent as *PRSS3* knockdown, consistent with attribution of the inhibitory activity on invasion with mesotrypsin inhibition. Consistent with the expectation of diminazene on inhibition of PRSS3-induced proliferation, no significant additional attenuation of proliferation was observed in PRSS3 KD cells (Supplemental Fig. 3a). A yet greater suppressive effect on invasion was seen with a diminazene concentration of 100 µM; this observation may reflect a role for other serine protease targets of diminazene in LAC invasion. In MTT assays, similar concentration-dependent inhibitory effects of diminazene were observed (Fig. 3b). Significant inhibition of cell growth was found at 10 µM diminazene; however, a concentration >100 µM was required to achieve similar effect to *PRSS3* knockdown.

### Kallikrein 5 (KLK5) is a potential mediator of mesotrypsin-promoted malignancy and is associated with poor survival and cancer progression in lung adenocarcinoma

**Figure 4:**
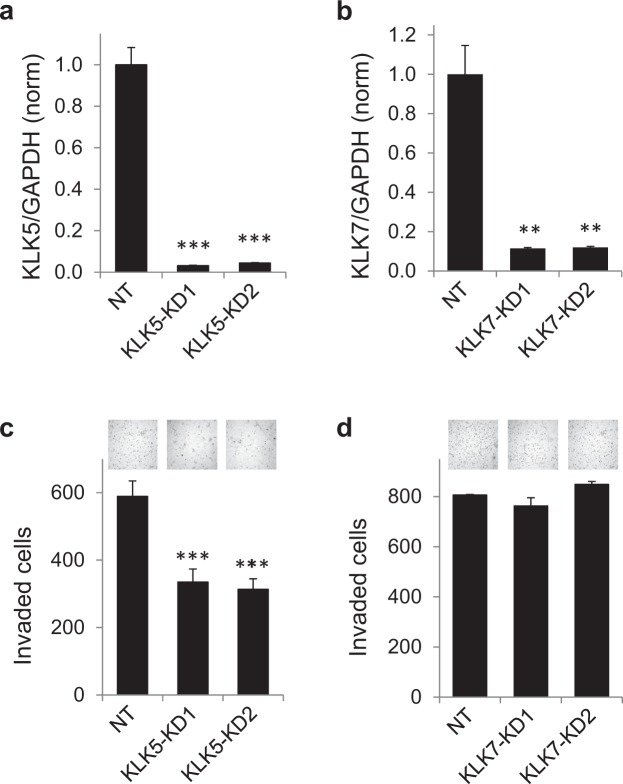
*KLK5* silencing inhibits invasion of PC9 lung adenocarcinoma cells. (**a**) *KLK5* expression is effectively suppressed by two targeted lentiviral shRNA constructs, KLK5-KD1 and KLK5-KD2, relative to cells transduced with the non-target control virus (NT), as assessed by qRT/PCR normalized to GAPDH expression. (**b**) *KLK7* expression is effectively suppressed by two targeted lentiviral shRNA constructs, KLK7-KD1 and KLK7-KD2, relative to cells transduced with the non-target control virus (NT), as assessed by qRT/PCR normalized to GAPDH expression. (**c**) Knockdown of *KLK5* significantly inhibits PC9 cellular invasion in Matrigel transwell assays. (**d**) By contrast, knockdown of *KLK7* has no significant impact on PC9 cellular invasion in Matrigel transwell assays. Representative images of the invasion assays are shown above each condition. Error bars show SEM. **P ≤ 0.01; ***P ≤ 0.001.

Mesotrypsin has been found to proteolyze as substrates and compromise the inhibitory activity of a variety of endogenous serine protease inhibitors that regulate the activities of multiple serine proteases relevant to cancer progression^17,19,25–27^. One family of serine proteases found to be widely upregulated in cancer is the human kallikrein family^13,14^. A survey of the literature identified human kallikrein-related peptidases 5 and 7 as enzymes expressed in lung cancer and potentially involved in malignant processes^13,14,28,29^. Intriguingly, mesotrypsin appears to be capable of directly activating pro-KLK5 and pro-KLK7 to their active forms, in addition to degrading the inhibitors of these proteases^27^. We hypothesized that mesotrypsin-promoted lung cancer cell invasion and growth may be mediated at least in part through the activation of kallikrein-related peptidases and the destruction of their endogenous inhibitors, resulting in increased activity of KLKs in the tumor microenvironment. To evaluate the functional roles of human kallikrein-related peptidases 5 and 7 in the PC9 cell model of LAC, we used lentiviral shRNA knockdown constructs targeting the *KLK5* and *KLK7* genes to specifically suppress their expression, and assessed the impact of the knockdowns on cellular invasion and growth. While both genes were expressed in PC9 cells and each was effectively silenced by lentiviral shRNA transduction (Fig. 4a,b), only *KLK5* knockdown produced consistent and significant effects in phenotypic assays. Knockdown of *KLK5* expression in PC9 cells led to significant reductions in cellular invasion in Matrigel transwell assays (Fig. 4c), similar to the effect seen upon knockdown of *PRSS3* in these cells (Fig. 2c), whereas knockdown of *KLK7* expression had no significant impact on cellular invasion (Fig. 4d; Western blot validations of knockdowns of KLK5 and KLK7, Supplemental Fig. 1b,c). Knockdown of *KLK5* expression also significantly inhibited cell growth of PC9 cells as measured using MTT assays (Supplemental Fig. 4), further mimicking the effect of *PRSS3* knockdown (Fig. 2d).

**Figure 5:**
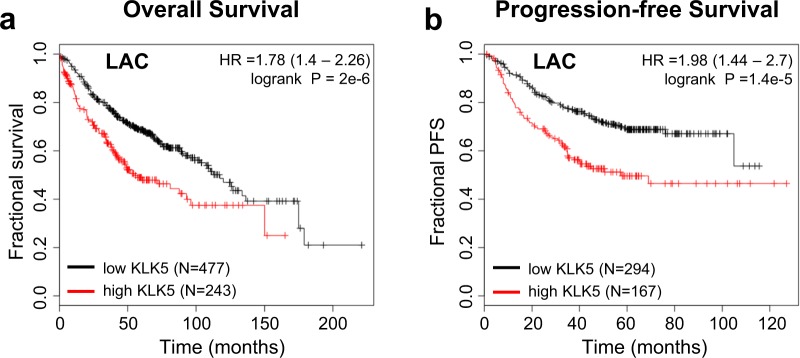
*KLK5* is prognostic of poor survival and cancer progression in lung adenocarcinoma (LAC). Univariate Kaplan-Meier survival analyses of LAC patients are plotted, stratified by *KLK5* mRNA expression, showing association of high *KLK5* expression with (**a**) poor OS and (**b**) poor PFS. Analyses were conducted using the KM Plotter online tool (http://kmplot.com/analysis/)^23^ and included data pooled from 10 cohorts; additional details are described in the Materials and Methods.

Having found that *KLK5* knockdown phenocopied the effects of *PRSS3* knockdown on cancer cell invasion and proliferation (Fig. 4, Supplemental Fig. 4), we next evaluated the potential association of *KLK5* gene expression in LAC with survival and progression. We found that high *KLK5* expression, like high *PRSS3* expression (Fig. 1**)**, was strongly associated with both poor OS **(**Fig. 5a**)** and poor PFS **(**Fig. 5b**)**. Together, these observations are consistent with the hypothesis that mesotrypsin promotion of cancer cell invasion and proliferation might be mediated through KLK5.

### *PRSS3* and *KLK5* drive expression of highly overlapping gene sets with roles in cancer progression

**Figure 6:**
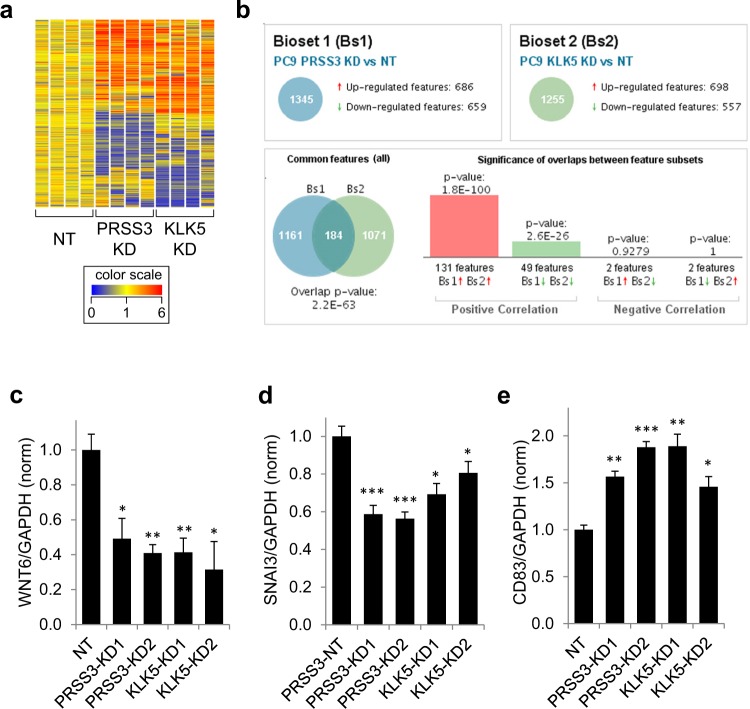
*PRSS3* and *KLK5* drive expression of highly overlapping gene sets in lung adenocarcinoma cells. (**a**) Heat map of 2416 genes differentially expressed between NT (control), PRSS3 KD, and KLK5 KD (P < 0.05, FC > 1.5). (**b**) Overlap of significantly differentially expressed genes (P < 0.05, FC > 1.5; N = 184) between PRSS3 KD vs NT (N = 1345) and KLK5 KD vs NT (N = 1255). (**c**) Knockdown of PRSS3 or KLK5 significantly decreases expression of WNT6. (**d**) Knockdown of PRSS3 or KLK5 significantly decreases expression of SNAI3. (**e**) Knockdown of PRSS3 or KLK5 significantly increases expression of CD83.

To further define the processes affected by the mesotrypsin-KLK5 signaling axis in LAC, we performed transcriptional microarray profiling of PC9 cells transduced with either *PRSS3* or *KLK5* knockdown viruses compared to control cells transduced with a non-target (NT) virus. Using a threshold of p < 0.05 and fold-change greater than 1.5, we identified 1357 genes that were significantly differentially expressed between *PRSS3* knockdown cells and NT control cells (Supplemental Table 1), and 1278 genes that were significantly differentially expressed between *KLK5* knockdown cells and NT control cells (Supplemental Table 2). Hierarchical clustering of these gene sets revealed substantial overlap between genes affected by *PRSS3* knockdown and *KLK5* knockdown (Fig. 6a), consistent with our expectation that *PRSS3* and *KLK5* are serial components of a critical signaling pathway. We analyzed gene set overlap using Illumina Correlation Engine, which identified 184 genes that were found in both significantly differentially expressed datasets (p for overlap = 2.2E-63; Fig. 6b). We selected three of the genes in the overlapping set for validation by qRT/PCR: *WNT6*, encoding a ligand of the WNT signaling pathways that are critical for lung cancer progression^30^ and which has been specifically associated with chemoresistance and poor prognosis in several cancer types^31–33^; *SNAI3*, which encodes the transcription factor SNAIL3 that drives epithelial-mesenchymal transition and consequent tumor cell metastasis and resistance to chemotherapy^34,35^; and *CD83*, encoding a dendritic cell surface protein studied extensively for its role in lymphocyte function^36^, but which also plays a critical role in immune cell function when expressed by thymic epithelial cells^37^ and additionally has been found to be expressed by lung epithelial and lung cancer cells^38^, where it may play a role in immunosurveillance. We found that *WNT6* and *SNAI3* were both significantly downregulated by *PRSS3* knockdown and *KLK5* knockdown (Fig. 6c,d), and that CD83 was significantly upregulated by PRSS3/mesotrypsin knockdown and KLK5 knockdown (Fig. 6e). We performed a meta-analysis to assess the association of *WNT6*, *SNAI3*, and *CD83* expression with lung cancer patient prognosis, and found that *WNT6* and *SNAI3*, which were downregulated with *PRSS3* and *KLK5* knockdown (Fig. 6c,d), were both found to be associated with better prognosis for LAC patients with lower expression of these genes (Supplemental Fig. 5), while *CD83*, which was upregulated with *PRSS3* and *KLK5* knockdown (Fig. 6e), was found to be associated with better prognosis for LAC patients with higher expression of this gene (Supplemental Fig. 6). Also consistent with hypothesized role of these molecules in a specific mesotrypsin-KLK5 oncogenic signaling process, the prognostic association was found specifically for patients with LAC, and not SCC (Supplemental Figs 5 and 6), similar to the prognostic association with *PRSS3* (Fig. 1).

## Discussion

Here, we have presented experiments implicating a PRSS3/mesotrypsin-KLK5 signaling axis in LAC malignancy. We showed that knockdown or inhibition of PRSS3/mesotrypsin inhibits LAC cancer cell invasion and proliferation, and that knockdown of KLK5 also inhibits LAC cancer cell invasion and proliferation. Through meta-analysis of published datasets, we identified tumor expression of *PRSS3* and *KLK5* transcripts as prognostic of poor survival and cancer progression in LAC but not SCC, which indicates that the pathways studied may be specific to LAC. We further found that down-modulation of either *PRSS3* or *KLK5* expression in LAC cells resulted in a robust transcriptional response with striking overlap between the *PRSS3* and *KLK5* regulated gene sets, implicating *PRSS3* and *KLK5* in a common signaling pathway in LAC. Given the extremely high significance of overlap between *PRSS3* and *KLK5* gene signatures, it is likely that that these genes, and the proteases they encode, may function together, and we hypothesize that mesotrypsin regulates the activity of KLK5 through mechanisms described in greater detail below. However, our attempts to detect modulation of KLK5 enzyme activity in PC9 cell cultures after *PRSS3* knockdown or mesotrypsin treatment produced inconclusive results (not shown), due to the inadequate enzyme selectivity of available reporter substrates. Nevertheless, our functional assays determined that both *PRSS3* and *KLK5* expression are important for LAC cell growth and invasion, and further, that inhibition of mesotrypsin activity can suppress LAC cell growth and invasion; thus, targeting the PRSS3/mesotrypsin-KLK5 regulatory axis may be of therapeutic relevance.

While our study did not directly define the nature of the mechanistic link between mesotrypsin and KLK5, it is common for proteases to act coordinately in cascades and networks, and there are several likely mechanisms by which mesotrypsin may influence the activity of KLK5. The most direct influence of mesotrypsin on KLK5 activity may derive from direct proteolytic activation; a previous report demonstrated that recombinant mesotrypsin can cleave recombinant pro-KLK5 *in vitro* to produce active KLK5^27^. A second mechanism by which mesotrypsin may influence the activity of KLK5 is through proteolytic inactivation of endogenous serine protease inhibitors that regulate KLK5 *in vivo*. KLK5 activity is known to be regulated by tight binding to several proteinaceous inhibitors, including lympho-epithelial Kazal-type inhibitor (LEKTI) encoded by *SPINK5*^39^, serine protease inhibitor Kazal-type 6 (SPINK6)^40^, and serine protease inhibitor Kazal-type 9 (SPINK9)^41^. Hepatocyte growth factor activator inhibitor 1 (HAI-1) likewise inhibits KLK5^42^, and the related Kunitz-type inhibitor HAI-2, formerly known as “placental bikunin”, also strongly inhibits kallikrein-related peptidases^43^, although its activity against KLK5 specifically has not been reported. All of these inhibitors of the Kazal and Kunitz families employ a common mechanism, using a conformationally defined canonical binding loop to occupy the catalytic site of target enzymes while resisting proteolysis^44,45^. Mesotrypsin recognizes this canonical binding loop as a substrate rather than an inhibitor, and cleaves the loop to render the inhibitor much less potent toward other target proteases^17,25^. Mesotrypsin is known to cleave and inactivate inhibitor domains of LEKTI^27^ and HAI-2^17^, and based on structural and mechanistic similarities among the inhibitors, may target other KLK5 inhibitors as well.

A second as-yet unresolved question concerns the downstream targets of KLK5 (and perhaps mesotrypsin) through which oncogenic signaling may be mediated in LAC. One possibility is signaling through protease-activated receptors (PARs), membrane-spanning G protein-coupled receptors that can be activated for signaling by proteolytic unmasking of a tethered ligand sequence^46^. KLK5 has been found to preferentially activate PAR2^47,48^, a receptor that is upregulated in LAC relative to normal alveolar tissue, and contributes to production of pro-inflammatory and pro-angiogenic cytokines^49,50^. Mesotrypsin can also signal through PARs in some settings, although with lesser potency than other trypsins; the ability of mesotrypsin to signal through PAR1 and PAR2 in epithelial tissues remains somewhat controversial^51,52^. Another route through which KLK5 may mediate oncogenic signaling is via proteolytic activation of hepatocyte growth factor activator (HGFA), a serine protease which in turn activates hepatocyte growth factor/scatter factor (HGF/SF), which binds to its receptor, the tyrosine kinase MET, to initiate a wide variety of malignant cellular alterations^53^. KLK5 has been identified as an efficient activator of latent pro-HGFA to active HGFA^42^. Mesotrypsin has not been reported as a direct activator of HGFA or HGF/SF, but may indirectly regulate this pathway through inactivation of the serine protease inhibitor HAI-2 (and perhaps HAI-1)^17^; in addition to inhibiting KLK5 as described above, these inhibitors are potent regulators of HGFA, hepsin, and matriptase, all serine proteases that activate pro-HGF/SF to active HGF/SF^12,53^.

The discovery of mesotrypsin and KLK5 as proteases involved in LAC progression presents the possibility of targeting the activities of these proteases for therapeutic benefit in LAC. The serine protease family has been estimated to represent around 4% of the “druggable genome”, comprised of human genes encoding proteins able to bind to drug-like molecules^54^. A recent analysis of molecular targets for approved drugs reveals proteases (of serine and other mechanistic classes) to represent a major target class responsible for the activity of 3.4% of drugs, but also a target class with unusually high attrition of candidate compounds in the course of drug development^55^. These statistics reflect the challenges associated with identifying efficacious targets, given the highly complex crosstalk among multiple proteases and endogenous protease inhibitors functioning in networks^15^, and also reflect the difficulty of developing selective drugs for individual protease targets within large, highly homologous enzyme families. While no currently approved drugs target either mesotrypsin or KLK5, recent research efforts have taken preliminary steps toward identifying selective pharmacological inhibitors of these proteases. For mesotrypsin, small molecule inhibitors identified have so far been limited to relatively weak binders that are poorly selective among trypsin isoforms^24,56^. An alternative approach aims to develop highly selective biologics targeting mesotrypsin, through protein engineering of the human amyloid precursor protein Kunitz protease inhibitor domain (APPI)^57^. Recent studies employing a powerful directed evolution approach to screen large libraries of APPI variants have resulted in marked improvements in mesotrypsin affinity, selectivity, and proteolytic stability^58,59^, lending encouragement that continued efforts may ultimately achieve inhibitors of therapeutic utility. Development of selective KLK5 inhibitors has likewise proven challenging given the structural similarity among multiple KLKs, but recent inhibitor development programs demonstrate ongoing interest in the pursuit of KLK5 as a potential drug target for skin diseases and cancer^60–64^. If these efforts succeed, it may become possible to therapeutically target mesotrypsin, KLK5, or a combination of these proteases to halt LAC progression.

## Materials and Methods

### Kaplan-Meier survival analyses

The data used in Figs 1 and 5, Supplemental Figs 5, 6 and Tables 1 and 2 are derived from 13 lung cancer cohorts deposited in the caArray project of the Cancer Biomedical Informatics Grid (http://cabig.cancer.gov/), the Gene Expression Omnibus (GEO, http://www.ncbi.nlm.nih.gov/geo/), and The Cancer Genome Atlas (TCGA, http://cancergenome.nih.gov). Accession numbers for cohorts included in analyses for each figure panel are as follows: 1A (CaArray, GSE14814, GSE19188, GSE29013, GSE30219, GSE31210, GSE3141, GSE31908, GSE37745, GSE4573, GSE50081, TCGA); 1B (CaArray, GSE29013, GSE31210, GSE31908, GSE50081, GSE8894); 1C and 5A (GSE14814, GSE19188, GSE29013, GSE30219, GSE31210, GSE3141, GSE31908, GSE37745, GSE50081); 1D and 5B (GSE29013, GSE31210, GSE31908, GSE50081, GSE8894); 1E (GSE14814, GSE19188, GSE29013, GSE30219, GSE3141, GSE37745, GSE4573, GSE50081, TCGA); 1F (GSE29013, GSE50081, GSE8894). All of the data were mined through the web tool Kaplan-Meier Plotter (http://kmplot.com/analysis/)^23^. Analyses presented for *PRSS3* expression in Fig. 1 and Tables 1 and 2 used the 213421_x_at Affymetrix probe; very similar results were found in parallel analyses (not shown) using the 207463_x_at Affymetrix probe. Analyses presented for *KLK5* expression in Fig. 5 used the 222242_s_at Affymetrix probe. Analyses presented in Supplemental Fig. 5 used the 71933_at Affymetrix probe for *WNT6* and the 1560228_at Affymetrix probe for *SNAI3*. Analyses presented in Supplemental Fig. 6 used the 204440_at Affymetrix probe for *CD83*. For all analyses, the “autoselect” mode was used to determine the optimal cut-off for high and low gene expression. A filtering option was selected to exclude potentially biased arrays as determined using several quality control measures^23^.

### Cell culture

The PC9 lung adenocarcinoma cell line was a kind gift from Dr. Christine Lovly, Vanderbilt University School of Medicine, Nashville, TN USA, and has been previously characterized in detail^65–69^. Short tandem repeat (STR) genotyping (PowerPlex® 16 HS platform; Promega) of the cell line stock following expansion gave a 100% match to the PC9 cell line profile reported in the DSMZ database (http://www.dsmz.de). Cell cultures initiated from this validated stock were passaged and used in experiments for up to three months. PC9 cells were maintained in RPMI 1640 (ATCC 30-2001) supplemented with 10% fetal bovine serum (FBS) (Gemini Bio-Products, Sacramento, CA, USA) and 1% penicillin-streptomycin (MP Biomedicals, Santa Ana, CA, USA). All cell cultures were grown at 37 °C in a humidified atmosphere with 5% CO_2_.

### Lentiviral shRNA knockdowns

Lentiviral shRNA constructs NM_002771.2-454s1c1 (PRSS3-KD1) and NM_002771.3-337s21c1 (PRSS3-KD2) targeting human *PRSS3*; NM_012427.31248s1c1 (KLK5-KD1) and NM_012427.3650s1c1 (KLK5-KD2) targeting the human kallikrein-related peptidase 5 gene (*KLK5*); and NM_005046.2-423 (KLK7-KD1) and NM_005046.2-990s1c1 (KLK7-KD2) targeting the human kallikrein-related peptidase 7 gene (*KLK7*) were from the MISSION TRC-Hs 1.0 and 1.5 libraries (Sigma, St. Louis, MO). A non-target control (NT) shRNA that does not recognize any human genes was used as a negative control in all RNAi experiments. Conditioned media containing infective lentivirus particles were produced using HEK 293FT cells following supplier protocols. For lentiviral transduction of PC9 cells, 1.5 × 10^6^ cells were seeded in 10 cm^2^ culture dishes. After 24 h, the medium was replaced with a mixture of 3.6 ml fresh RPMI medium containing 10% FBS and 10 µg/mL polybrene (Fisher) plus 2.4 mL of lentiviral particle-containing conditioned medium. The medium was changed again after 24 h and transduced cells were selected with 2 µg/mL puromycin (Corning, Kennebunk, ME). Western blot validation of knockdown was performed using (for PRSS3) our previously characterized mesotrypsin antibody^20,57^, (for KLK5) Novus Biologicals #NB200-138 (Centennial, CO), and (for KLK7) R&D Systems #AF2624 (Minneapolis, MN).

### RNA extraction cDNA synthesis, and quantitative real-time PCR

RNA was isolated using TRIzol reagent (Invitrogen) according to manufacturer protocols, and was converted to cDNA using the High Capacity cDNA Reverse Transcription Kit (Applied Biosystems, Foster City, CA) according to kit specifications. Quantitative real-time PCR was performed using TaqMan gene expression assays (Applied Biosystems) on an Applied Biosystems 7900HT Fast Real-Time PCR system according to manufacturer protocols. TaqMan assays employed included: GAPDH (Hs99999905_m1), PRSS3 (Hs00605637_m1), KLK5 (Hs01548153_m1) and KLK7 (Hs00192503_m1). Data were analyzed using SDS RQ Manager Software (Applied Biosystems).

### Invasion assay

Invasion assays followed a protocol similar to those we have described previously^20,70^. PC9 cells were split at 1:2.5 the day before the assay. BD Biocoat control 24-well inserts (8.0 microns) were coated with 50 µg of Matrigel basement membrane matrix in 100 µL of serum-free RPMI and incubated at 37 °C for 4 h. Cells were rinsed with cold 1× PBS and then lifted with 0.25% trypsin, and then suspended in 1 mL of RPMI media containing 0.1% BSA and counted using a Countess automated cell counter (Invitrogen). 1 × 10^5^ cells were seeded into each insert in a final volume of 500 µL, while the bottom chambers contained 750 µL of NIH/3T3 cell conditioned medium (serum-free DMEM supplemented with 50 µg/mL ascorbic acid) as chemo-attractant. Quadruple replicates were set per condition. Cells were allowed to invade for 18 h at 37 °C in 5% CO_2_. Noninvading cells were removed from the insert by scrubbing with a cotton swab, and then invaded cells were fixed with 100% methanol for 30 minutes at −20 °C and finally, stained with 0.1% crystal violet in 20 mM MES (pH 6.0) for 1 h at room temperature. Filters were dried at room temperature, photographed with 20× magnification, and counted with Image-Pro 6.3 software (Media Cybernetics). Consistent results were obtained from four independent experiments.

### MTT viability assay

To measure cell proliferation/viability, 6 replicates of 5000 cells per well were plated in 96 well plates in 100 µL complete media, grown 24 h, and then incubated with 20 µL of 5 mg/mL 3-(4,5-dimethylthiazol-2-yl)-2,5-diphenyl tetrazolium bromide (MTT, Amresco) at 37 °C for 4 h according to the manufacturer protocol. Supernatants were aspirated and formazan crystals were dissolved in 100 µL DMSO for 2–4 h at 37 °C and then absorbance at 540 nm was measured using a FlexStation3 plate reader using SoftMax Pro 7.0 software (Molecular Devices). Graphed data show average and SEM of biological replicates.

### Transcriptional microarray profiling and analysis

Lentiviral knockdown of *PRSS3* and *KLK5* in PC9 cells using each of the NT control virus, two *PRSS3*-targeted shRNA constructs, and two *KLK5*-targeted shRNA constructs was conducted in duplicate experiments, and RNA extraction was performed for each experiment, as described above. RNA was assessed with Affymetrix human U133_Plus_2 gene expression chips and processed and analyzed using GeneSpring 13.0, as described previously^71^. Genes significantly affected by *PRSS3* knockdown (n = 1357) were identified as FC > 1.5 and p < 0.05 of the four knockdown samples (two different knockdown constructs in duplicate) by comparison with NT control samples (n = 4). Genes significantly affected by *KLK5* knockdown (n = 1357) were identified as FC > 1.5 and p < 0.05 of the four knockdown samples (two different knockdown constructs in duplicate) by comparison with NT control samples (n = 4). Gene overlap was performed using Correlation Engine (Illumina) as described previously^70^. Gene expression profiles have been deposited in the Gene Expression Omnibus (GSE112785 is the superseries containing all expression data; GSE112783 is the KLK5 knockdown expression subseries; GSE112784 is the PRSS3 knockdown expression subseries).

## Supplementary information

Supplementary Figures

Supplemental Table 1

Supplemental Table 2

## Data Availability

Gene expression profiles have been deposited in the Gene Expression Omnibus (GSE112785 is the superseries containing all expression data; GSE112783 is the KLK5 knockdown expression subseries; GSE112784 is the PRSS3 knockdown expression subseries).
