# Supplementary material for: PRSS3/Mesotrypsin and kallikrein-related peptidase 5 are associated with poor prognosis and contribute to tumor cell invasion and growth in lung adenocarcinoma
Source: Sci Rep. 2019 Feb 12;9:1844. doi: 10.1038/s41598-018-38362-0 (PMC6372636; doi:10.1038/s41598-018-38362-0)
Supplement: Supplementary file 1 — Supplementary Figures [file 41598_2018_38362_MOESM1_ESM.pdf]

Supplemental Figures

PRSS3/Mesotrypsin and kallikrein-related peptidase 5 are associated with poor prognosis and contribute to tumor cell invasion and growth in lung adenocarcinoma

Honghai Ma[1,2], Alexandra Hockla[1], Christine Mehner[1], Matt Coban[1], Niv Papo[3], Derek C. Radisky[1*], and Evette S. Radisky[1*]

[1]Department of Cancer Biology, Mayo Clinic, Jacksonville, FL 32225, USA
[2]Department of Thoracic Surgery, First Affiliated Hospital, School of Medicine, Zhejiang University, Hangzhou 310003, China
[3]Department of Biotechnology Engineering and the National Institute of Biotechnology in the Negev, Ben-Gurion University of the Negev, Beer-Sheva, Israel

Supplemental Figure 1

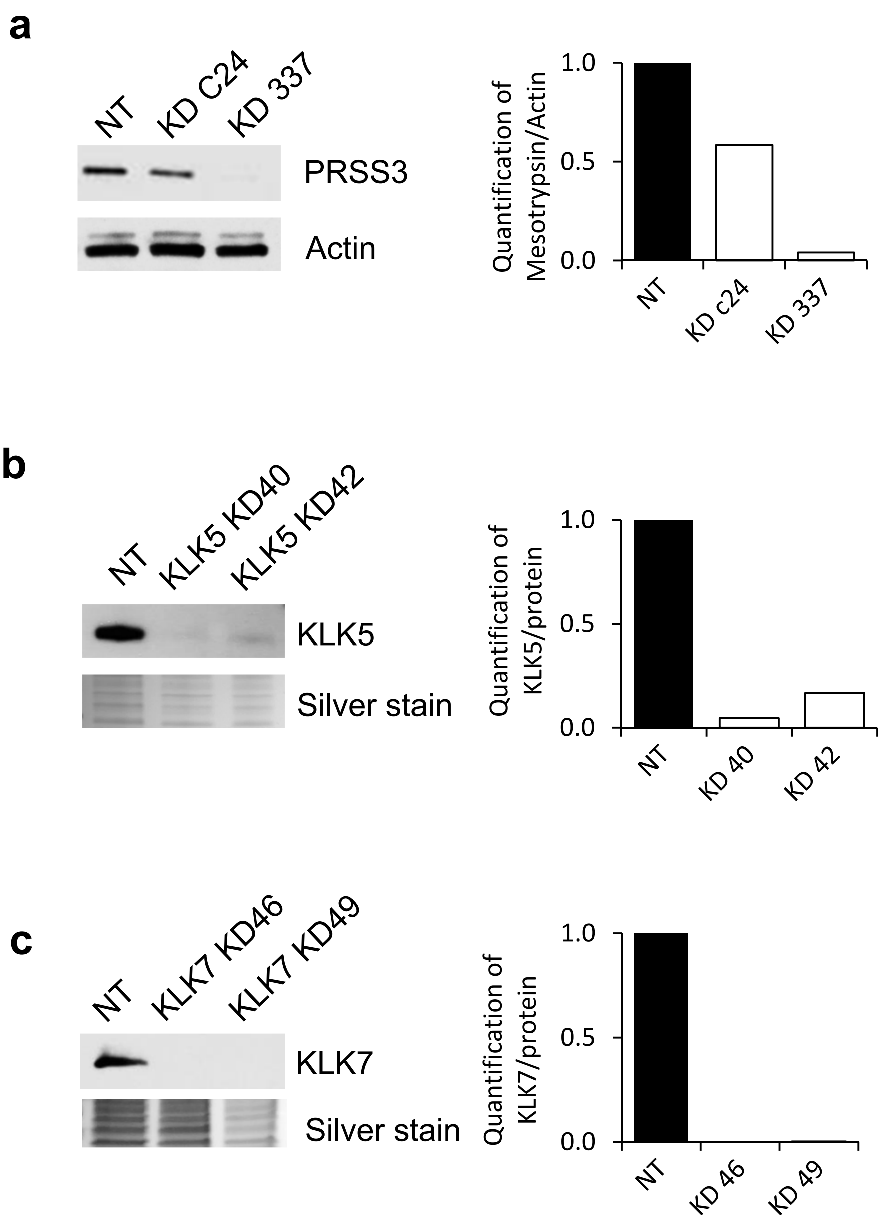

Supplemental Figure 1. Western blot efficiency of PRSS3-KD, KLK5-KD, and KLK7-KD in PC9 cells. Western blots (left) and quantification of blots (right) are shown for nontarget (NT) and two knockdown constructs. (a) PRSS3 knockdown comparing lysates from NT, and PRSS3-specific KD c24 (designated as PRSS3-KD1 in the main figures) and KD 337 (designated as PRSS3-KD2 in the main figures). (b) KLK5 knockdown comparing conditioned media from NT, and KLK5-specific KD 42 (designated as KLK5-KD1 in the main figures) and KD 42 (designated as KLK5-KD2 in the main figures). (c) KLK7 knockdown comparing conditioned media from NT, and KLK7-specific KD 46(designated as KLK7-KD1 in the main figures) and KD 49 (designated as KLK7-KD2 in the main figures).

Supplemental Figure 2

a

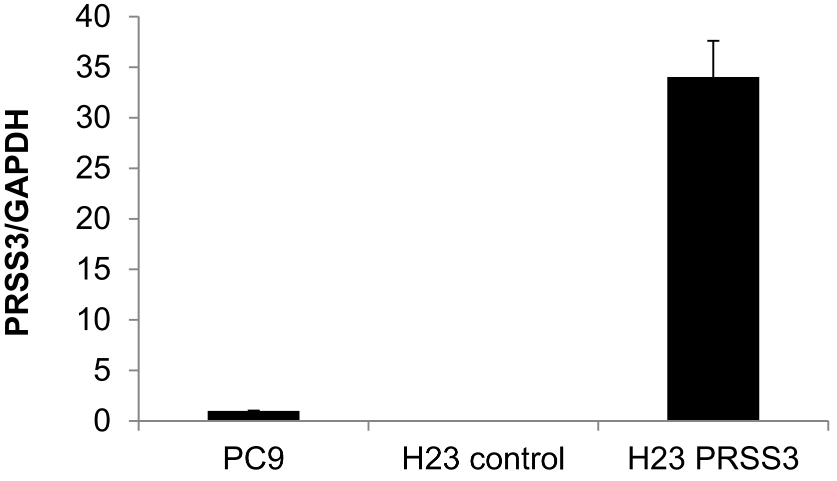

b

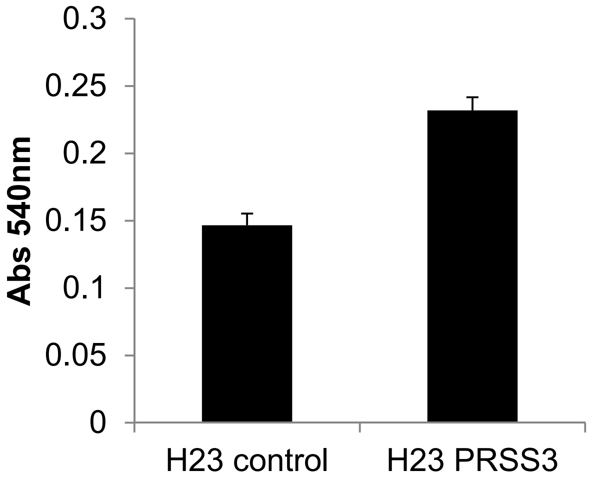

Supplemental Figure 2. Overexpression of PRSS3 leads to increased proliferation in H23 cells. (a) Quantitative PCR of PRSS3 expression in PC9 cells, and in H23 cells expressing control vector (H23 control) or vector encoding PRSS3 (H23 PRSS3). **(b)** Assessment of proliferation by MTT assay in response to PRSS3 overexpression. Error bars show SEM. P<0.005 for MTT assay comparison.

Supplemental Figure 3

a

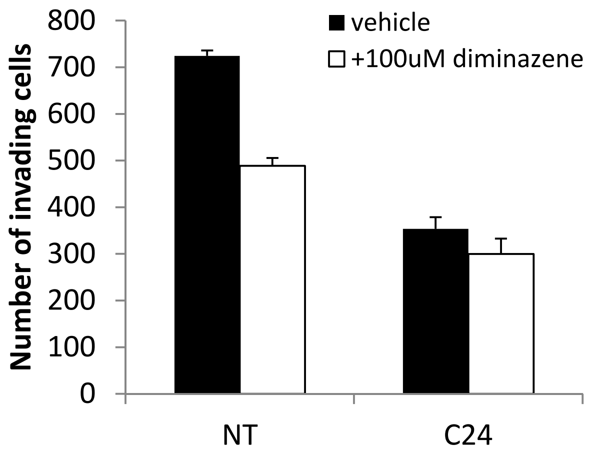

b

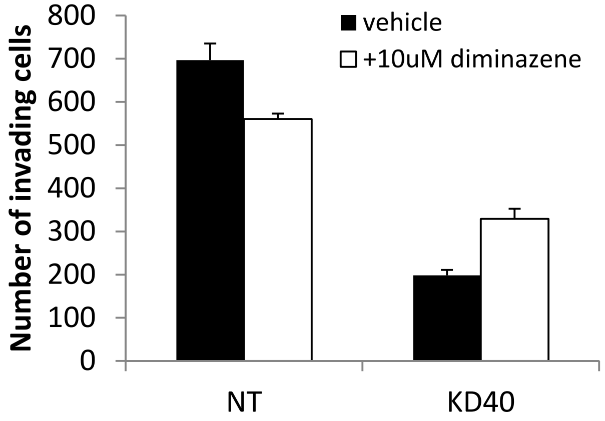

Supplemental Figure 3. Diminazene treatment shows no additional inhibition of cell invasion with knockdown of PRSS3 or KLK5. Invasion assay results for knockdown of PRSS3 **(a)** and KLK5 **(b)**, with and without treatment with diminazene. Error bars show SEM.

Supplemental Figure 4

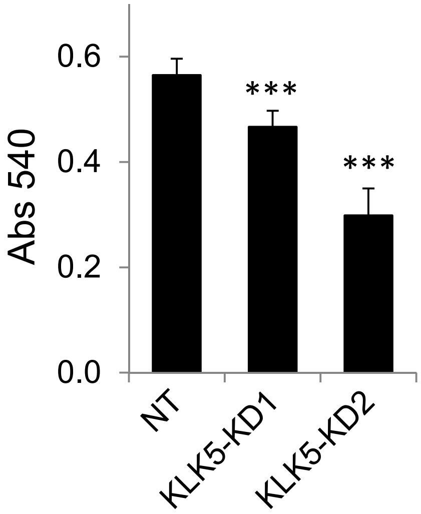

Supplemental Figure 4. KLK5 silencing inhibits growth of PC9 lung adenocarcinoma cells. Knockdown of KLK5 significantly inhibits PC9 cell growth as assessed by MTT assays. Error bars show SEM. ***, P<0.001

Supplemental Figure 5

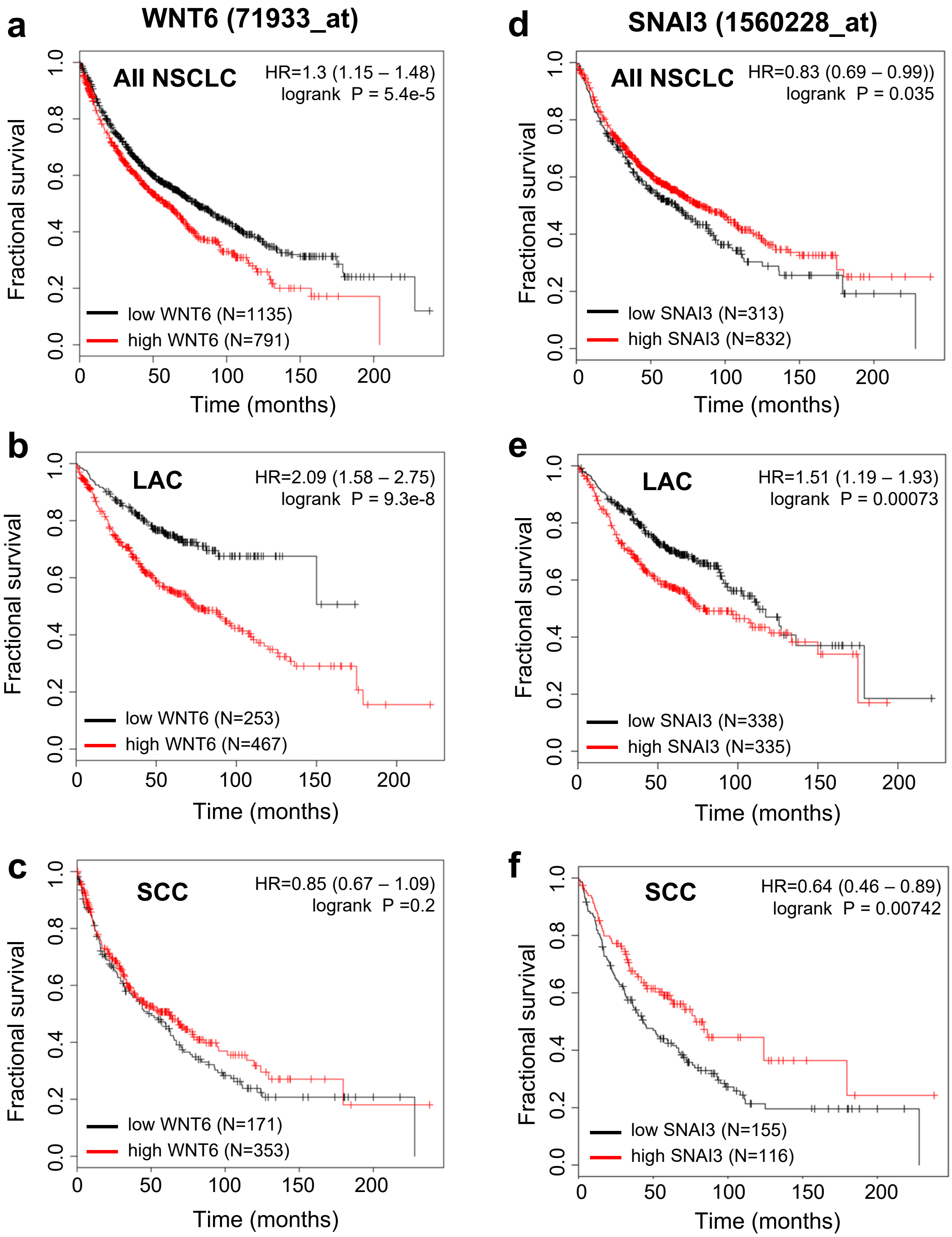

Supplemental Figure 5

Supplemental Figure 2. WNT6 and SNAI3, genes whose expression are reduced by knockdown of PRSS3 or KLK5 (Fig. 6c,d), are prognostic of poor survival in lung adenocarcinoma (LAC) but not squamous cell carcinoma (SCC). Univariate Kaplan-Meier survival analyses of NSCLC patients are plotted, stratified by WNT6 or SNAI3 mRNA expression. **(a)** High WNT6 expression was significantly associated with poor OS in analyses including all NSCLC patients. **(b)** Analyses restricted to patients with LAC showed yet stronger significant association of WNT6 expression with poor OS. **(c)** Analyses of the subset of patients with SCC did not show significant association of WNT6 expression with clinical outcomes. **(d)** High SNAI3 expression was weakly associated with better OS in analyses including all NSCLC patients. **(e)** Analyses restricted to patients with LAC showed strong and significant association of SNAI3 expression with poor OS. **(f)** Analyses of the subset of patients with SCC showed an opposite, significant association of SNAI3 expression with better OS. Analyses were conducted using the KM Plotter online tool (http://kmplot.com/analysis/) and included data pooled from 13 cohorts; additional details are described in the Materials and Methods.

Supplemental Figure 6

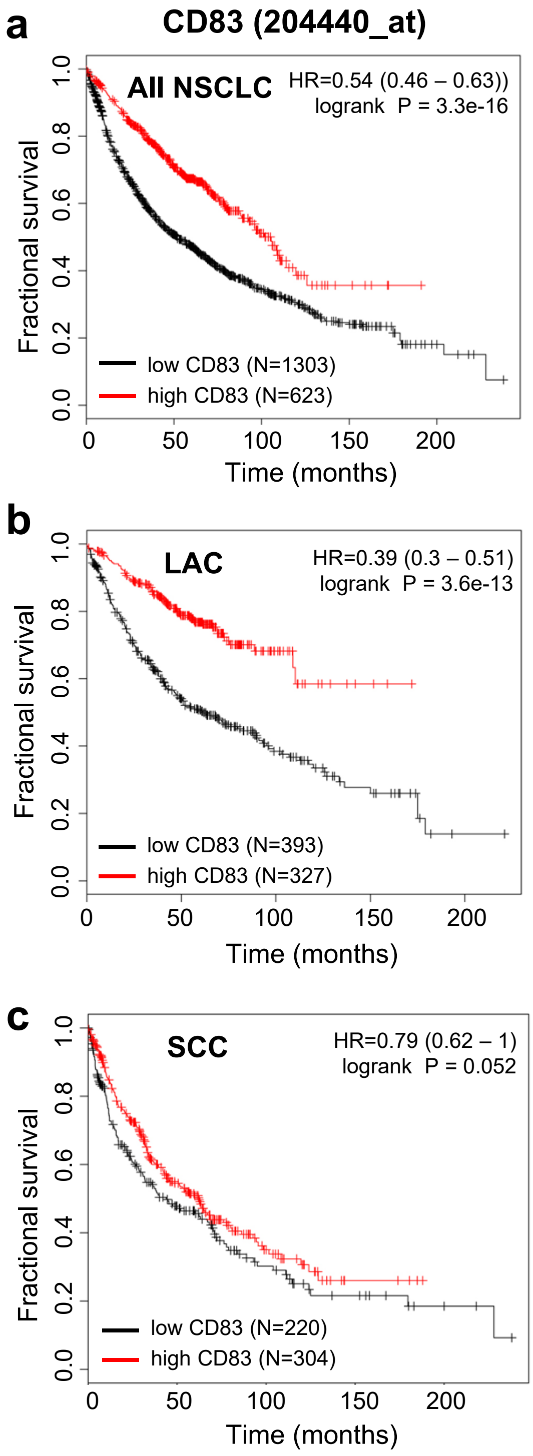

Supplemental Figure 6. CD83, which shows increased expression by knockdown of PRSS3 or KLK5 (Fig. 6e), is prognostic of better survival in lung adenocarcinoma (LAC) but not squamous cell carcinoma (SCC). Univariate Kaplan-Meier survival analyses of NSCLC patients are plotted, stratified by CD83 mRNA expression. **(a)** Low CD83 expression was significantly associated with poor OS in analyses including all NSCLC patients. **(b)** Analyses restricted to patients with LAC showed yet stronger significant association of low CD83 expression with poor OS. **(c)** Analyses of the subset of patients with SCC did not show significant association of CD83 expression with clinical outcomes. Analyses were conducted using the KM Plotter online tool (http://kmplot.com/analysis/) and included data pooled from 13 cohorts; additional details are described in the Materials and Methods.
